# Temporal Analysis of the Associations Between Radiological Imaging Prevalence and Life Expectancy at Birth and Mortality

**DOI:** 10.3390/diagnostics16213513

**Published:** 2026-10-27

**Authors:** İsmail Kartal, Kadir Yılmaz

**Affiliations:** 1 Radiology, Ordu City Hospital, Ordu 52200, Türkiye; 2 Department of Statistics, Istanbul Commerce University, İstanbul 34445, Türkiye; dekartezyen@hotmail.com

**Keywords:** radiological imaging, life expectancy at birth, mortality

## Abstract

**Background/Objectives**: This study aimed to analyze the temporal associations between radiological imaging prevalence and life expectancy at birth and mortality. **Methods**: The study used the *Türkiye Ministry of Health Statistics Yearbook*, World Health Organization (WHO) and World Bank data to analyze radiological imaging counts, female life expectancy at birth, and female mortality levels for Turkey between 2015 and 2023. The widest date range from both datasets was selected for the analysis. Because the Ministry of Health Statistics provides the widest date range for radiological imaging as 2015–2023, the research dataset was limited to this date range. **Results**: There was no direct relationship between radiologic imaging and life expectancy at birth or female mortality (*p* > 0.05). Year-controlled partial correlation analysis results showed that life expectancy at birth was significantly correlated with CT imaging numbers (r = −0.854; *p* < 0.01). MR, CT, ultrasound, Doppler, ECO and mammography had no significant association with female life expectancy at birth (*p* > 0.05). Similarly, associations between MR, CT, ultrasound, Doppler, ECO and mammography and female mortality were statistically insignificant (*p* > 0.05) **Conclusions**: In the study, no univariate association reached *p* < 0.05; the single nominal finding for CT is negative and unstable, and the analysis is exploratory.

## 1. Introduction

Early diagnosis is especially crucial for diseases with high mortality rates, such as cancer [1,2]. Many types of cancer can be treated through early diagnosis, allowing for both improved quality of life for patients [3] and reduced healthcare burden [4]. Radiologic methods are used as the standard diagnostic method for the majority of diseases with high mortality. Diagnostic radiology, in general, encompasses imaging and diagnostic methods based on radiation technology, used in many fields, particularly cancer [5,6]. Common applications of Computed Tomography (CT), X-ray, ultrasound, Magnetic Resonance Imaging (MRI), and nuclear medicine form the basis of radiologic diagnostics [7]. These systems are dynamic and progressive, evolving daily with new technological advances and adapting to emerging technologies such as artificial intelligence [8]. These systems are multidisciplinary structures, encompassing not only the medical field but also medical engineering and information systems.

In diagnostic radiology, the very name “radiation” and its underlying principle have been the subject of debate from past to present. There are studies suggesting that screenings such as mammography, particularly in healthy individuals, have no benefit and, on the contrary, carry high potential for harm [9,10]. However, studies suggesting this also report that radiation diagnosis, despite its population drawbacks, offers significant disease-related benefits [11,12]. Numerous studies on mortality through radiological diagnosis have been conducted suggesting that early diagnosis reduces mortality. These include breast cancer [9,10,11,12], lung cancer [13,14], ileus [15], and even asymptomatic myosteatosis, which is often aided by a wide range of factors. While significant advances have been made in radiologic imaging devices and techniques in recent years, efforts are ongoing to minimize their harmful effects. Despite this, there remains a prevailing bias toward radiologic devices and imaging methods, as well as concerns about their potential harms. Despite persistent public concern and radiophobia regarding potential radiation exposure [11], diagnostic imaging methods remain indispensable for the emergency triage and management of patients with critical non-oncological conditions such as acute ischemic stroke, myocardial infarction, and severe trauma [15,16]. Consequently, when evaluating the widespread adoption of imaging techniques [6], it is essential to weigh common public misconceptions against the tangible clinical benefits that these methods provide in diagnosing conditions associated with high mortality rates [1,13].

While population screening remains debated due to overdiagnosis, targeted diagnostic imaging is essential for managing high-mortality diseases. In these cases, the clinical benefits of rapid triage and staging must be balanced against long-term stochastic radiation risks. Addressing public concerns regarding radiation safety therefore requires evidence-based communication. While there are studies focusing on this topic with small clinical samples, there are insufficient studies examining the topic quantitatively on a larger scale and at a population level. Therefore, demonstrating the overall associations through population-based studies could help encourage clinicians and patients. Therefore, this study aimed to analyze the temporal associations between radiological imaging prevalence and life expectancy at birth and mortality.

## 2. Materials and Methods

### 2.1. Research Model

This study is strictly exploratory and hypothesis-generating, not confirmatory. First, using time-series and longitudinal analysis, changes in the number of radiological imaging data, female mortality, and female life expectancy at birth for Türkiye were analyzed over time. The relationship between radiological imaging series and these variables and their associations were then analyzed.

The dynamics of the indicators within our nine-year time series were significantly influenced by non-linear external shocks, primarily the COVID-19 pandemic. The simultaneous decline in the volume of imaging procedures (MRI, ultrasound, Doppler imaging, echocardiography, mammography) and the spike in mortality during 2020–2021 (with life expectancy dropping to 79.00 years in 2021) represent shared consequences of this singular exogenous shock rather than the result of a direct causal link. Furthermore, at the population level, the model is subject to confounding by indication: the rise in demand for imaging could be driven by an aging population or an increase in the baseline disease burden. To evaluate the potential disproportionate impact of the 2020–2021 period on the baseline trends, a univariate sensitivity analysis was performed by excluding these two years (*n* = 7). The complete statistical outputs, including 95% confidence intervals, are detailed in Supplementary Table S1. The analysis demonstrated that the statistical non-significance observed at baseline remained largely stable for most imaging modalities.

### 2.2. Research Data

The study involved the use of *Türkiye Ministry of Health Statistics Yearbook*, World Health Organization (WHO) and World Bank data to analyze radiological imaging counts, female life expectancy at birth, and female mortality levels for Turkey between 2015 and 2023. The widest date range from both datasets was selected for the analysis. Since the Ministry of Health provides the widest date range for radiological imaging of 2015–2023, the research dataset was limited to this date range. All radiological imaging scans performed and reported to the Ministry of Health each year were included in the study. The dependent and independent variables used in the study were as follows:

Dependent variables:

- Life expectancy at birth, female (years);
- Mortality rate, adult, female (per 1000 female adults).

Independent variable:

- Number of imaging procedures per 1000 people in hospitals.

When selecting research data, proportional data was used instead of raw data. This aimed to minimize confounder associations. Mortality and number of views were presented as rates per 1000 women. The variables of the research are given in Table 1.

All data used in the analysis were obtained from the relevant institution’s latest updated data release. It must be noted that these parameters represent modeled demographic estimates rather than directly observed raw vital statistics. Although female life expectancy at birth remained unchanged between 2016 and 2023 (79.86 years), a noticeable shift was observed in adult female mortality rates (53.21 versus 64.99 per 1000 adults). Within the limits of the aggregate national dataset, this mathematical discrepancy between stable life expectancy and fluctuating adult mortality remains statistically unexplained, as a formal age-decomposition analysis could not be performed. These series are sourced from the World Bank Open Data (World Development Indicators, 2024 Release), utilizing indicator codes SP.DYN.LE00.FE.IN for female life expectancy and SP.DYN.AMRT.FE for adult female mortality. In obtaining patient imaging data, the figures from the ministry’s official website and reports were used. Accordingly, the count was based not on a per-patient basis, but rather on the ratio of the total number of imaging procedures to the total number of patients.

In this study, the exposure variable was defined and analyzed as the ‘number of imaging procedures per 1000 people in hospitals’. The denominator encompasses the general hospital population, while the numerator is based on the total number of imaging procedures performed in hospitals; these data were obtained from ministry reports. The data series reflects the entire hospital ecosystem, covering both private sector and university hospitals. Among the outcome variables analyzed, adult female mortality was measured as the ‘number of deaths per 1000 adult women’; this data was sourced from the WHO.

To address the serious risks associated with temporal dependence and spurious correlations driven by common macro-level time trends (where independent variables appear linked simply because both exhibit monotonic growth over time), two distinct strategies were employed. First, we conducted a partial correlation analysis controlling for year to isolate non-linear residuals by removing the common linear chronological trend. Second, given that employing complex parametric methods to correct for non-linear disturbances was not feasible, we refrained from drawing any far-reaching causal or predictive conclusions. The manuscript explicitly characterizes all identified relationships solely as descriptive and exploratory instances of co-movement, while emphasizing that measures of nominal significance are highly sensitive to temporal artifacts and the impact of the 2020–2021 pandemic shock.

Before assessing pairwise or multifactorial relationships between the frequency of imaging studies and public health indicators, we conducted a baseline analysis of time-series dynamics to identify the underlying temporal trajectories of each variable. In historical macro-level data, variables often exhibit a pronounced monotonic upward or downward trend driven solely by the passage of time and independent long-term factors such as population aging, technological progress, and the expansion of healthcare system capacity. Analyzing relationships without first identifying these chronological patterns carries a high risk of spurious correlation, where a statistical link between entirely independent indicators appears to exist merely due to the coincidence of their temporal vectors. This preliminary analysis of dynamics enabled us to clearly distinguish these linear trends and isolate the significant non-linear shift caused by the shock of the COVID-19 pandemic in 2020–2021, prior to constructing our analytical models.

The selection of imaging modalities was based on their prevalence in clinical practice and their standardization within Ministry of Health reporting systems; particular emphasis was placed on advanced techniques of proven public health significance. In addition to comprehensive modalities such as CT and MRI, specialized techniques (e.g., Doppler ultrasound and echocardiography) were also selected, enabling a representative overview of the most widely used diagnostic tools.

Instead of ‘raw’ frequency figures, normalized and standardized rates were used; this made it possible to adjust for changes in population size over the nine-year period under review. The use of standardized metrics—such as the number of imaging procedures per 1000 people served by a hospital—ensures the mathematical comparability of data across different years by neutralizing the effect of rising absolute volumes.

### 2.3. Ethical Approval

The data used in the study comprised the number of radiological images taken within the entire population and did not include patient personal or demographic data. Therefore, the research data is publicly available in the *Türkiye Ministry of Health Statistical Yearbooks*, WHO and World Bank databases (Table 1). Therefore, because the research data is public and there are no patients to directly obtain consent from, ethics committee approval and informed patient consent are not applicable.

### 2.4. Statistical Methods

Parity analysis was performed for the time series analysis of changes in images taken according to radiological imaging types, female life expectancy at birth, and female mortality parameters. Mean, standard deviation, median, and variation ranges were used to define the study parameters. Spearman’s rank correlation coefficient was selected as the primary measure of pairwise association instead of the Pearson correlation coefficient. The small sample size (*n* = 9) inherently limits the applicability of asymptotic parametric assumptions. Since the Spearman’s rho coefficient operates on ranks rather than raw values, it provides a robust, distribution-independent, and outlier-resistant assessment of non-linear monotonic relationships. Furthermore, this study was conducted and reported in strict alignment with the Strengthening the Reporting of Observational Studies in Epidemiology (STROBE) statement guidelines. Due to linearization deviations [17], Linear Model analysis was performed for regression analysis. SPSS 25.0 for Windows was used for the analyses. All analyses were performed with an upper limit of significance level of 0.05 and a 95% confidence interval. Multiple regression modeling was entirely abandoned due to severe multicollinearity (VIF > 200), an excessively high matrix condition number (>40,000), and limited degrees of freedom (residual df = 2) observed in the multivariate saturated model. Since these parametric anomalies rendered the interpretation of individual coefficients for the independent variables impossible, our analysis strategy was shifted to a series of univariate Ordinary Least Squares (OLS) linear regression models. Using this approach, the isolated effect of each imaging modality was tested individually, thereby increasing the degrees of freedom to 7 (df = 7) and correcting for variance inflation.

Regarding the issue of multiple comparisons and the exploratory nature of the study, a total of 48 formal hypothesis tests were conducted based on the same 9 annual observations including analyses of pairwise and partial correlations, as well as the estimation of slope coefficients via ordinary least squares regression. Given the small sample size (*n* = 9) and the performance of multiple statistical assessments, the authors explicitly acknowledge the risk of an inflated Type I error rate. To ensure statistical rigor and avoid overinterpreting results that border on statistical significance, all analytical procedures in this article are classified as strictly exploratory and hypothesis-generating rather than confirmatory. In accordance with Armstrong’s criteria, no formal adjustments for multiple comparisons (such as the Bonferroni correction or false discovery rate control) were applied to artificially achieve statistical significance [18]; instead, we openly report all unadjusted *p*-values while refraining from formulating clinical or organizational recommendations applicable to the general population. Forty-eight tests were run on the same nine annual observations. As no single global null hypothesis is tested and the analysis is exploratory, no correction was applied [1]; all *p*-values are unadjusted and descriptive.

## 3. Results

In 2015, rates per 1.000 patients were 143 for MR, 174 for CT, 348 for ultrasound, 138 for Doppler, 88 for ECO and 25 for mammography. In 2023, rates per 1.000 patients were 222 for MR, 314 for CT, 232 for ultrasound, 306 for Doppler, 108 for ECO and 32 for mammography. All radiologic imaging rates were increased from 2015 to 2023 except USG, and USG was decreased. Ultrasound decreased from 348 to 232 per 1000 between 2015 and 2023. Life expectancy at birth was 79.74 year in 2015, and increased to 79.86 in 2023. The female mortality rate was 53.81 per 1.000 female adults in 2015, and reached 64.99 in 2023 (Table 2).

**Figure 1:**
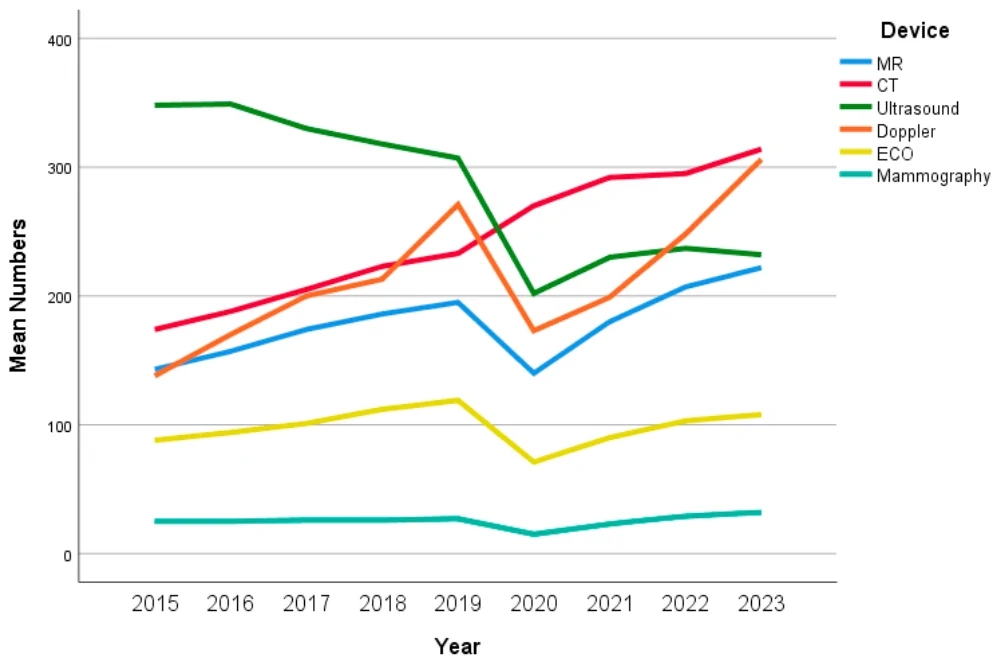
Radiologic imaging number changes according to years.

In 2020, all radiologic imaging numbers except CT were decreased due to the COVID-19 pandemic, and afterwards, an increasing trend was recorded. Following the 2020–2021 pandemic period, a pronounced increasing trend in absolute volumes was observed for advanced modalities such as CT and MRI; however, this upward trajectory did not hold for ultrasound examinations, which remained relatively stable (Figure 1).

The imaging mean values were 178.22 ± 27.97 for MR, 243.78 ± 50.74 for CT, 283.67 ± 57.74 for ultrasound, 213.11 ± 53.24 for Doppler, 98.44 ± 14.48 for ECO, and 25.33 ± 4.66 for mammography. Life expectancy at birth for females ranged from 79 years to 80.89 years with 80.09 ± 0.59 mean. Female mortality per 1.000 female adults ranged from 47.84 to 64.99 with 53.58 ± 5.98 mean value (Table 3).

**Figure 2:**
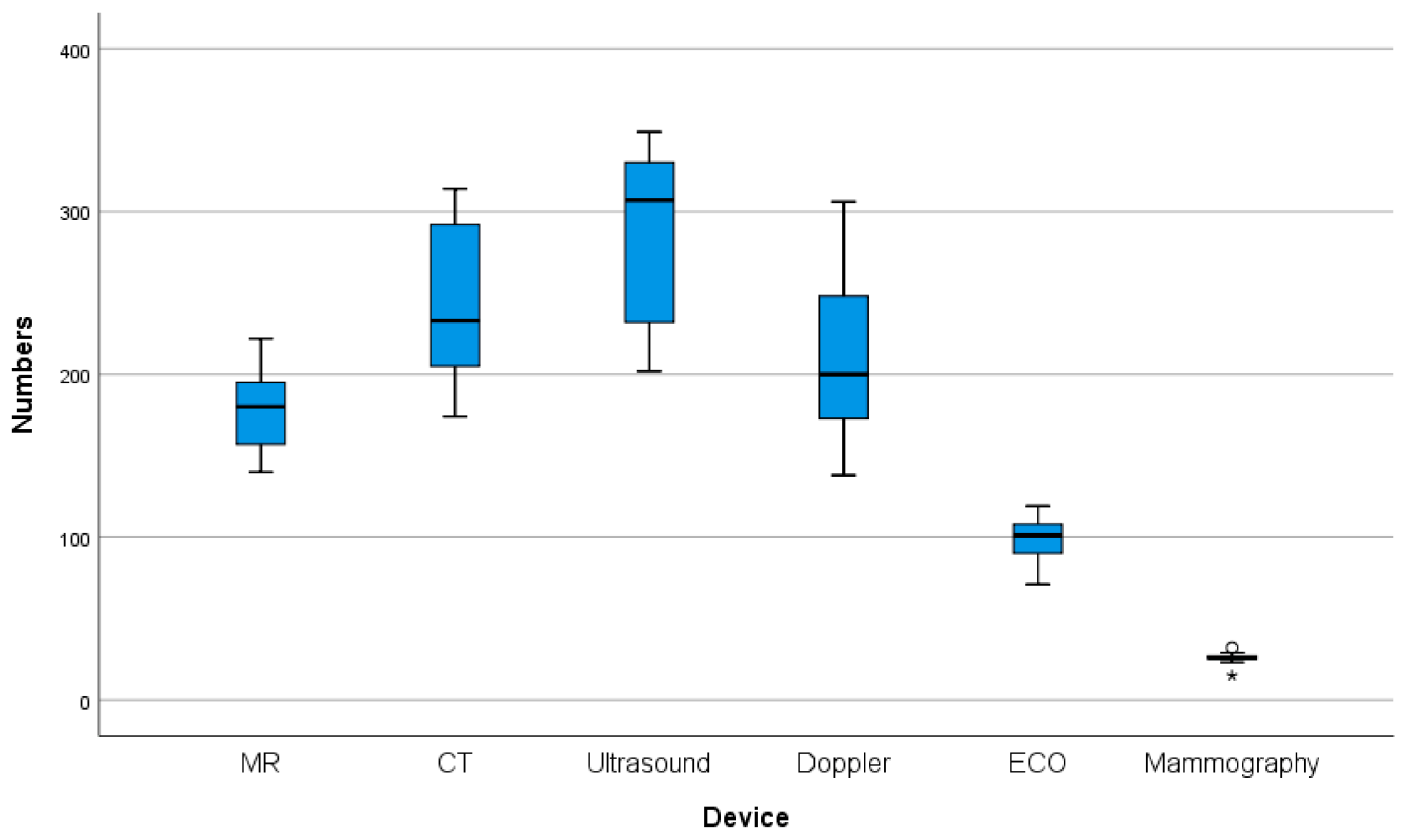
Mean radiologic imaging values and ranges for 2015–2023 time period.

Ultrasound imaging medians and interquartile ranges (IQRs) were the highest, followed by CT, Doppler, MR, ECO and mammography (Figure 2).

The Spearman’s rho correlation analysis results showed that there was no direct relationship between radiologic imaging and life expectancy at birth or female mortality (*p* > 0.05) (Table 4).

The year-controlled partial correlation analysis results showed that life expectancy at birth was significantly correlated with CT imaging numbers (r = −0.854; *p* < 0.01). For all other imaging types, there was no direct relationship with life expectancy at birth or female mortality (*p* > 0.05) (Table 5).

Univariate linear model analysis results showed that MR, CT, ultrasound, Doppler, ECO and mammography had no significant associations with female life expectancy at birth (*p* > 0.05). Similarly, the associations between MR, CT, ultrasound, Doppler, ECO and mammography and female mortality were statistically insignificant (*p* > 0.05) (Table 6).

## 4. Discussion

This study aimed to analyze the temporal associations between radiological imaging prevalence and life expectancy at birth and mortality, using data from 2015 to 2023 for Turkey on a population basis. The results showed that among all radiological imaging types and numbers, only CT had a correlation with female life expectancy at birth.

Early diagnosis is vital in the treatment of many diseases with high mortality rates, especially cancer [1,4], and radiation imaging is generally one of the most basic and reliable methods in the diagnosis of such diseases [7,8]. Furthermore, because radiological imaging methods essentially operate through radiation mechanisms and because radiation has negative effects on health, some studies in the literature suggest that radiological screening may do more harm than good [9,10,11,12]. While there is a common consensus in the literature that radiological imaging methods are important for early diagnosis in patient groups, there is criticism of screening the general population. However, it is theoretically impossible to diagnose a patient group that already has a diagnosis. Therefore, although international health authorities publish guidelines and directives on radiological screening, there are still studies that approach the subject with negative perspectives [9,10,11].

Cancer types are particularly prominent in the evaluation of the benefits and harms of radiological imaging methods. Among these, Grimm et al. [19] reported that the health risks for breast cancer patients due to radiation-induced malignancy and excessive anxiety are insignificantly lower than those associated with early diagnosis with radiological imaging. Hooshmand et al. [20] similarly reported that the reduction in breast cancer mortality is more valuable than the radiation-induced risks. Mok et al. [21] reported that interventional radiology has a significant effect on mortality and morbidity. Hall and Brenner [22] reported that although the use of CT is increasing in asymptomatic patients, a comprehensive evaluation of its effects is necessary. Hall [23] pointed out that the diagnostic value and benefit of radiological imaging for an individual patient is higher, but that its risk may increase when applied to the entire population. Linet et al. [24] reported that instead of limiting the potential harms of diagnostic radiation techniques, it is necessary to demonstrate their effects based on evidence and to reduce them. Faulkner [25], in his study comparing the risks and benefits of diagnostic radiology, reported that the benefits of radiological diagnosis are vital and indispensable.

The fundamental conclusion emerging from these studies is that radiologic diagnosis, and especially early diagnosis, is crucial for patient groups. Therefore, there is no disagreement regarding radiologic diagnosis in patient groups. However, the issue of screening in healthy individuals is more complex. Within this context, the aggregate macro-level trends observed in our study should be interpreted strictly as exploratory metrics of healthcare utilization across Turkey, rather than definitive evidence of direct causal contributions to mortality outcomes or unmeasured clinical indicators such as patient quality of life.

To ensure the full transparency and reproducibility of our analysis, the complete raw data (unrounded)—specifically, the precise values for female life expectancy at birth—have been provided as Supplementary Materials. When constructing the initial Spearman rank correlation matrix, the data for 2016 and 2023 (nominally rounded to 79.86 years) were treated as independent ranks based on their differences in their raw (floating-point) form rather than being assigned averaged ranks for identical values.

Research variables such as life expectancy, mortality, and radiological imaging can be influenced not only by health indicators, but also by numerous confounders, including economic and social indicators, disease prevalence, and the overall health status of the population. Therefore, detailed studies addressing these confounders could be conducted in future research.

Although early diagnosis is critical for diseases with high mortality rates as it enables intervention at a localized stage and improves survival outcomes, the scientific literature raises concerns: it is argued that the mass radiological screening of asymptomatic populations often leads to significant overdiagnosis, undue anxiety, and unacceptable cumulative radiation risks that outweigh the marginal benefit to the population as a whole. In the present study, striking a balance between these conflicting viewpoints is significantly complicated by the study’s aggregated population-level design, which constitutes our primary limitation. Because the unit of analysis is the calendar year rather than individual patient data, it is impossible to directly correlate specific clinical indications with survival outcomes; consequently, the relationships observed at the macro level are susceptible to the “ecological fallacy” and must be interpreted solely as preliminary, hypothesis-generating trends rather than as causal mechanisms.

A longitudinal analysis was conducted using baseline variables despite their aggregate limitations because mapping chronological trajectories over time is a mandatory diagnostic step to identify structural shifts, such as the non-linear 2020 pandemic anomaly. However, the scope of this time series remains strictly bounded by a date-related constraint, as 2023 represents the final official vintage of fully audited public health and mortality statistics available at the time of this investigation. To build upon these boundaries and enhance generalizability, future research must transition to individual-level cohort designs with secure record-linkage systems, incorporating patient-specific clinical indications and a comparative male data series to definitively overcome the ecological fallacy.

Assessing these standardized data at the population level helps resolve ongoing debates surrounding radiological screening by providing a neutral, macro-level baseline of temporal trends, effectively distinguishing underlying chronological growth from sudden exogenous shocks, such as the pandemic. Ultimately, these findings contribute directly to clinical practice not by establishing definitive causal guidelines, but by serving as a preliminary alert to clinicians regarding the need to account for baseline variability in nominal statistics and the high risk of population-level data distortion driven by clinical indications.

However, the association between echocardiography (ECO) and female life expectancy at birth achieved statistical significance upon omitting the 2020–2021 cohort (B = 0.034, *p* = 0.047, 95% CI: [0.001, 0.067]). This indicates that the 2020–2021 data points may have exerted an attenuating effect on certain healthcare utilization metrics rather than artificially driving the overall baseline models.

To isolate fundamental interrelationships against the backdrop of long-term chronological trends, we conducted a partial correlation analysis adjusted for the year factor. The observed mathematical discrepancy in the relationship between CT volume and life expectancy—specifically, the shift from a non-significant Spearman rank correlation coefficient (r = 0.067; *p* = 0.865) to a strong negative partial correlation (r = −0.854; *p* = 0.007) after accounting for the calendar year—is a direct artifact of the linear detrending procedure. Since both CT volumes and life expectancy exhibit strong, mutually independent linear upward trends over time, removing the effect of the calendar year eliminates this shared temporal variation. The resulting high negative coefficient reflects residual non-linear covariation within the context of a very small sample size (*n* = 9; effective degrees of freedom df = 5). Consistent with the objectives of our exploratory analysis, this sharp shift is interpreted not as evidence of a robust population-level causal link, but rather as an indicator of the statistical instability inherent in small-sample time series when statistical control methods are applied.

### 4.1. Limitations of the Study

The most significant limitation of this study is the limited number of confounders, as it is a population-based study. Because no previous studies addressing radiological imaging in a similar manner were found in the literature, a longitudinal analysis was conducted using baseline variables with limitations. However, further research could enhance the generalizability of our findings by adding new confounders to our existing variables.

Another significant limitation stems from the dataset’s date range. The Ministry of Health’s *Statistical Yearbook* provides the most up-to-date validated information on radiological imaging for 2023. As stated previously, retrospective data from the period prior to 2015 were explicitly excluded because significant inconsistencies in institutional reporting and changes in clinical tracking protocols prior to that cutoff precluded reliable data standardization. Consequently, the analyzed timeline is structurally restricted to this nine-year period.

A key limitation of this study is its aggregated (ecological) design, in which the unit of analysis is the calendar year (*n* = 9) rather than the individual patient. Although data on the volume of radiological examinations are based on complete medical facility records for each year, such temporal aggregation precludes drawing conclusions about population-level processes at the individual level. Due to the inability to link data on specific patients who underwent examinations with outcomes (survival or mortality), the study results are susceptible to the “ecological fallacy.” Trends in examination frequency observed at the macro level cannot be causally linked to specific pathological processes, clinical indications, or individual patient management trajectories. Within the hierarchy of evidence, this analysis falls into the category of descriptive and exploratory research and serves solely to generate hypotheses based on observed co-variations in metrics over time.

Clinical classification, gender-related factors, and latency-related limitations: We openly acknowledge a number of significant clinical and structural limitations inherent in our data system. First, in most healthcare systems, echocardiography (ECHO) is inherently classified as a cardiology procedure rather than a radiological one, while Doppler ultrasound is considered a type of ultrasound examination rather than a distinct, independent category. The absence of standardized data regarding conventional radiography, fluoroscopy, and nuclear medicine modalities creates an unavoidable issue of data incompleteness. Second, there is a significant conceptual asymmetry: whereas mammography is a screening method intended exclusively for women within a specific age group, utilization rates for other imaging modalities encompass both sexes and all age categories. Restricting the metrics analyzed solely to mortality among the adult female population and female life expectancy at birth precludes a comparative analysis against an alternative scenario (counterfactual analysis); consequently, the lack of data on the male population is a recognized limitation that hinders broader extrapolation of the results. Finally, regarding the study’s objective—comparing the actual benefits of imaging modalities against the harms of radiation exposure—the nine-year follow-up period is biologically insufficient to assess the stochastic risks of radiation-induced malignancies, given that these conditions are characterized by a well-documented latency period ranging from one to several decades.

### 4.2. The Study’s Contributions to the Literature and Clinical Practice

In conclusion, this study offers an ecological overview of radiological imaging trends over a nine-year period. Given the limited sample size (*n* = 9) and the aggregate nature of the macro-level dataset, these findings should be interpreted as exploratory baseline metrics and do not support any definitive causal inferences. Furthermore, these short-term population-level trends do not investigate direct biological outcomes and are not intended to contradict or challenge the established epidemiological literature regarding long-term stochastic radiation risks. Future research utilizing individual-level longitudinal data is required to further elucidate the direct clinical impact of these imaging modalities.

This study provides a transparent description of national imaging volumes alongside female life expectancy and adult mortality in Türkiye, 2015–2023. Because the design is ecological, the series is short (*n* = 9), and no individual-level outcomes were available, the data cannot be used to infer benefit or harm of imaging.

## 5. Conclusions

In the study, no univariate association reached *p* < 0.05; the single nominal finding, CT, is negative and unstable; and the analysis is exploratory. For echocardiography and mammography, further studies are needed that include other confounders and disease prevalence and incidence. In summary, the population-level data over the reviewed period demonstrate that radiological imaging utilization rates changed heterogeneously across modalities, while adult female mortality rates experienced an upward trajectory and female life expectancy at birth remained essentially unchanged. For future research in echocardiography and mammography, it is recommended that additional clinical parameters—such as cohort age ranges, left ventricular ejection fraction (LVEF) values, histopathological monitoring data, and comparative data from male subjects—are considered in order to eliminate demographic asymmetry and the confounding of different Doppler techniques. Based on the final conclusions of this exploratory study, clinicians and patients are advised to approach diagnostic imaging methods with caution and rigorous clinical triage, remaining fully aware of the high risk of population-level “confounding by indication” and the instability of statistical trends observed in small samples.

## Figures and Tables

**Table 1:** Research parameters, data definitions, and institutional sources.

Parameter	Definition	Source	Exact Indicator Code/Dataset Version	URL and Access Date
MR	Number of imaging procedures per 1000 hospital patients	Türkiye Ministry of Health	*Health Statistics Yearbook* (Annual Editions, 2015–2023)	saglik.gov.tr Accessed: 12 October 2026
CT	Number of imaging procedures per 1000 hospital patients	Türkiye Ministry of Health	*Health Statistics Yearbook* (Annual Editions, 2015–2023)	saglik.gov.tr Accessed: 12 October 2026
Ultrasound	Number of imaging procedures per 1000 hospital patients	Türkiye Ministry of Health	*Health Statistics Yearbook* (Annual Editions, 2015–2023)	saglik.gov.tr Accessed: 12 October 2026
Doppler	Number of imaging procedures per 1000 hospital patients	Türkiye Ministry of Health	*Health Statistics Yearbook* (Annual Editions, 2015–2023)	saglik.gov.tr Accessed: 12 October 2026
ECO	Number of imaging procedures per 1000 hospital patients	Türkiye Ministry of Health	*Health Statistics Yearbook* (Annual Editions, 2015–2023)	saglik.gov.tr Accessed: 12 October 2026
Mammography	Number of imaging procedures per 1000 hospital patients	Türkiye Ministry of Health	*Health Statistics Yearbook* (Annual Editions, 2015–2023)	saglik.gov.tr Accessed: 12 October 2026
Life expectancy at birth	Female life expectancy at birth (years)	World Bank Open Data (*Compiled from WHO and UN*)	Indicator Code: SP.DYN.LE00.FE.IN (World Development Indicators)	data.worldbank.org Accessed: 12 October 2026
Female mortality	Adult mortality rate (per 1000 female adults)	World Bank Open Data (*Compiled from WHO and UN*)	Indicator Code: SP.DYN.AMRT.FE (World Development Indicators)	data.worldbank.org Accessed: 12 October 2026

Note: For health outcome indicators, data were cross-verified using the World Health Organization (WHO) Mortality Database (All Causes Explorer). MR: Magnetic Resonance, CT: Computed Tomography, ECO: echocardiography.

**Table 2:** Radiologic imaging numbers according to image type, life expectancy at birth, and female mortality for 2015–2023.

Year	MR ^a^	CT ^a^	USG ^a^	Doppler ^a^	ECO ^a^	Mammography ^a^	Life Expectancy at Birth ^b^	Female Mortality ^c^
2015	143	174	348	138	88	25	79.74	53.81
2016	157	188	349	170	94	25	79.86	53.21
2017	174	205	330	200	101	26	80.25	51.02
2018	186	223	318	213	112	26	80.63	49.12
2019	195	233	307	271	119	27	80.89	47.84
2020	140	270	202	173	71	15	79.89	50.39
2021	180	292	230	199	90	23	79.00	62.06
2022	207	295	237	248	103	29	80.72	49.82
2023	222	314	232	306	108	32	79.86	64.99

^a^: 1000 people in hospitals, ^b^: years, ^c^: per 1000 female adults, MR: Magnetic Resonance, CT: Computed Tomography, ECO: echocardiography.

**Table 3:** Descriptive statistics for radiologic imaging, life expectancy at birth and female mortality.

	Mean	Standard Deviation	Median	Minimum	Maximum
MR	178.22	27.97	180.00	140.00	222.00
CT	243.78	50.74	233.00	174.00	314.00
Ultrasound	283.67	57.74	307.00	202.00	349.00
Doppler	213.11	53.24	200.00	138.00	306.00
ECO	98.44	14.48	101.00	71.00	119.00
Mammography	25.33	4.66	26.00	15.00	32.00
Life expectancy at birth	80.09	0.59	79.89	79.00	80.89
Female mortality	53.58	5.98	51.02	47.84	64.99

MR: Magnetic Resonance, CT: Computed Tomography, ECO: echocardiography.

**Table 4:** Spearman’s rho correlation analysis for relationship between radiologic imaging and female life expectancy at birth and female mortality.

	Life Expectancy at Birth			Female Mortality		
	r	CI at 95%	*p*	r	CI at 95%	*p*
MR	0.350	−0.409, 0.823	0.356	−0.083	−0.708, 0.615	0.831
CT	0.067	−0.625, 0.700	0.865	0.117	−0.593, 0.725	0.765
Ultrasound	0.067	−0.625, 0.700	0.865	−0.067	−0.700, 0.625	0.865
Doppler	0.517	−0.224, 0.879	0.154	−0.250	−0.784, 0.497	0.516
ECO	0.650	−0.025, 0.918	0.058	−0.467	−0.863, 0.286	0.205
Mammography	0.479	−0.271, 0.867	0.192	−0.151	−0.741, 0.570	0.698

Note: 95% confidence intervals (CIs) for Spearman’s rho were computed using the robust Bonett–Wright transformation. MR: Magnetic Resonance, CT: Computed Tomography, ECO: echocardiography.

**Table 5:** Year-controlled partial correlation analysis for relationship between radiologic imaging and female life expectancy at birth and female mortality.

	Life Expectancy at Birth			Female Mortality		
	r	*p*	CI at 95%	r	*p*	CI at 95%
MR	0.524	0.182	−0.265, 0.893	−0.011	0.980	−0.670, 0.658
CT	−0.854 **	0.007	−0.976, −0.324	0.448	0.265	−0.343, 0.867
Ultrasound	0.474	0.235	−0.317, 0.876	0.046	0.915	−0.638, 0.689
Doppler	0.630	0.094	−0.134, 0.924	−0.128	0.763	−0.732, 0.588
ECO	0.653	0.079	−0.100, 0.930	−0.196	0.642	−0.764, 0.544
Mammography	0.346	0.402	−0.432, 0.829	0.200	0.635	−0.541, 0.766

** *p* < 0.01. Note: 95% confidence intervals (CIs) for Spearman’s rho were computed using the robust Bonett–Wright transformation. MR: Magnetic Resonance, CT: Computed Tomography, ECO: echocardiography.

**Table 6:** Linear regression for associations between radiologic imaging and female life expectancy at birth and female mortality at the univariate level.

	Unstandardized Coefficients		Standardized Coefficients	t	*p*	CI at 95%
	B	Std. Error	Beta			
Life expectancy at birth						
MR	0.008	0.007	0.361	1.024	0.340	−0.009, 0.025
CT	−0.001	0.004	0.128	−0.342	0.743	−0.011, 0.009
Ultrasound	0.002	0.004	0.237	0.645	0.540	−0.008, 0.012
Doppler	0.005	0.004	0.412	1.196	0.271	−0.005, 0.015]
ECO	0.026	0.012	0.645	2.232	0.061	−0.002, 0.054
Mammography	0.042	0.045	0.333	0.935	0.381	−0.064, 0.148
Female mortality						
MR	0.065	0.077	0.305	0.846	0.425	−0.117, 0.247
CT	0.058	0.039	0.491	1.492	0.179	−0.034, 0.150
Ultrasound	−0.038	0.036	−0.371	−1.056	0.326	−0.123, 0.047
Doppler	0.028	0.041	0.250	0.684	0.516	−0.069, 0.125
ECO	−0.046	0.155	−0.113	−0.300	0.773	−0.413, 0.321
Mammography	0.352	0.466	0.274	0.755	0.475	−0.750, 1.454

Note: This model was unadjusted for calendar year. MR: Magnetic Resonance, CT: Computed Tomography, ECO: echocardiography.

## Data Availability

The research data is publicly available in the *Türkiye Ministry of Health Statistical Yearbooks*, WHO and World Bank databases given in Table 1.

## Supplementary Materials

The following supporting information can be downloaded at: https://www.mdpi.com/article/10.3390/diagnostics16213513/s1, Table S1: Radiologic imaging numbers according to image type, life expectancy at birth, and female mortality for 2015–2023.

## Abbreviations

CT	Computerized Tomography
ECO	Echocardiography
MR	Magnetic Resonance
MRI	Magnetic Resonance Imaging
